# A Label-Free, Quantitative Fecal Hemoglobin Detection Platform for Colorectal Cancer Screening

**DOI:** 10.3390/bios7020019

**Published:** 2017-05-05

**Authors:** Gita V. Soraya, Thanh C. Nguyen, Chathurika D. Abeyrathne, Duc H. Huynh, Jianxiong Chan, Phuong D. Nguyen, Babak Nasr, Gursharan Chana, Patrick Kwan, Efstratios Skafidas

**Affiliations:** 1 Department of Medicine, Royal Melbourne Hospital, The University of Melbourne, Victoria 3050, Australia; gsoraya@student.unimelb.edu.au (G.V.S.); jianxiong.chan@unimelb.edu.au (J.C.); 2 Department of Biochemistry, Faculty of Medicine, Hasanuddin University, South Sulawesi 90245, Indonesia; 3 Centre for Neural Engineering, The University of Melbourne, Carlton, VIC 3053, Australia; nguyenct@student.unimelb.edu.au (T.C.N.); chathurika.abeyrathne@unimelb.edu.au (C.D.A.); d.huynh5@student.unimelb.edu.au (D.H.H.); pnguye@student.unimelb.edu.au (P.D.N.); babak.nasr@unimelb.edu.au (B.N.); gchana@unimelb.edu.au (G.C.); 4 Department of Electrical and Electronic Engineering, Melbourne School of Engineering, The University of Melbourne, Victoria 3010, Australia; 5 Department of Psychiatry, Royal Melbourne Hospital, The University of Melbourne, Victoria 3050, Australia

**Keywords:** biosensors, immunosensor, interdigitated electrodes, colorectal cancer, screening, impedance, point of care, label-free, diagnostics

## Abstract

The early detection of colorectal cancer is vital for disease management and patient survival. Fecal hemoglobin detection is a widely-adopted method for screening and early diagnosis. Fecal Immunochemical Test (FIT) is favored over the older generation chemical based Fecal Occult Blood Test (FOBT) as it does not require dietary or drug restrictions, and is specific to human blood from the lower digestive tract. To date, no quantitative FIT platforms are available for use in the point-of-care setting. Here, we report proof of principle data of a novel low cost quantitative fecal immunochemical-based biosensor platform that may be further developed into a point-of-care test in low-resource settings. The label-free prototype has a lower limit of detection (LOD) of 10 µg hemoglobin per gram (Hb/g) of feces, comparable to that of conventional laboratory based quantitative FIT diagnostic systems.

## 1. Introduction

Colorectal cancer (CRC) accounts for 10.0% and 9.2% of all cancers in men and women, respectively [1]. The annual number of new CRC cases has been forecasted to increase from 1.2 to 2.2 million cases worldwide within the next two decades [2]. Notably the majority of the rising incidence is expected to occur in developing countries [3,4], which has been attributed to the adoption of Westernized lifestyles and transition into chronic-degenerative disease dominated causes of mortality as these countries continue to undergo economic transition from low to a middle-income status [4].

Survival of CRC is highly dependent on the stage of diagnosis. Five-year survival ranges from 90% for CRC detected at the localized stage; 70% for regional; and down to 10% in people with distant metastasis [5]. Although colonoscopy remains the gold standard for CRC diagnosis [6], fecal occult blood test (FOBT) as means of detecting hemoglobin in the fecal sample is a valuable screening tool that has been incorporated into nation-wide screening programs in high income countries [7,8]. However, the administration of FOBT in low- to middle-income countries largely occurs on a case-to-case basis to triage colonoscopy referrals [9].

In general, there are numerous ways to detect forms and variants of hemoglobin, including enzymatic, cationic chromatography, affinity chromatography, and immunochemical methods [10]. However, the commonly used tests for detection of hemoglobin in fecal samples include the guaiac-based FOBT (gFOBT), and the fecal immunochemical test (FIT). The gFOBT utilizes guaiac and hydrogen peroxide to detect the heme component of hemoglobin. Although it is cheaper [11], gFOBT is less specific towards colorectal bleeding, requires subjective interpretation and is prone to both positive as well as negative interferences [12,13]. In comparison, through immunochemical based detection of the globin moiety, the FIT is more sensitive and specific towards colorectal bleeding [14]. In addition, unlike gFOBT, FIT does not require dietary restrictions, is less affected by concomitant medication use, and requires fewer stool samples.

Currently, FIT tests are available in qualitative and quantitative formats. The former is available in a point-of-care cassette-like format while the latter is laboratory based. They also differ in the way the cutoff value is set. Whilst cutoffs for qualitative FITs are pre-set by the manufacturers, quantitative FITs allow the user to set their desired cutoff value [15]. This provides flexibility to adjust cutoff values to suit local CRC screening policy. Quantitative FITs also allow the development of tailored risk algorithms for different subpopulations because fecal hemoglobin concentrations may be affected by age, gender, or geographical location [15,16,17]. Laboratory-based quantitative FIT has been shown to have higher sensitivity and specificity for CRC [18,19], and is beginning to replace gFOBT for CRC screening in high-income countries [12,20]. Table 1 compares the performance characteristics of qualitative and quantitative FIT products commonly used in CRC screening programs.

Despite its advantages over qualitative FIT, laboratory-based quantitative FIT is not practical in limited resource settings owing to infrastructural, geographical and financial constraints. To overcome these limitations, we have developed a novel, low cost and quantitative FIT biosensor platform adaptable into a point-of-care device. The biosensors detect or quantify biochemical molecules or proteins based on their binding affinities. The biosensors contain immobilized capture probes which can bind to the corresponding target molecule from a complex solution and result in a change at a localized surface. There are many methods to evaluate this change. Among them, the impedance biosensor allows quantification of biological molecules in a sample by measuring the changes in the capacitance or resistance [24] caused by the binding of target molecules to the immobilized probes [25]. The compact planar impedance biosensors can be implemented as part of integrated on-chip systems and require a smaller volume of sample for the measurements compared with laboratory based platforms, an essential property for point-of-care devices [24]. The interdigitated electrode (IDE) sensors are highly sensitive and have been used to perform label-free detections of a wide range of biological materials including DNA [26,27,28], antigen-antibody interactions [29,30,31], and whole cells [32,33,34,35].

Here, we report the development of surface optimized IDE sensors for quantitative detection of hemoglobin protein in human feces. The devices were transformed to electrochemical biosensors by immobilization of anti-hemoglobin antibody receptor on to the IDEs, which specifically binds to hemoglobin protein spiked in human feces. The binding caused a change in the device impedance at concentrations as low as 10 µg·Hb/g feces, comparable to the lower detection limit of conventional bench-top quantitative FIT detection systems (Table 1). The results provide proof-of-principle data that makes it feasible to quantitatively measure hemoglobin concentration in fecal samples using label-free impedance spectroscopy technology.

## 2. Materials and Methods

Ninety-nine percent (3-Aminopropyl)triethoxysilane (APTES), human hemoglobin (in lyophilized powder form) and rabbit polyclonal anti-human hemoglobin antibody (whole purified antiserum, product code H4890-2ML) were purchased from Sigma Aldrich (St Louis, MO, USA). 25% glutaraldehyde and 98% Ethanolamine were purchased from the University of Melbourne Chemical Store (Melbourne, Australia). Standard microscope slide (Menzel SuperFrost) was ordered from Thermo Fisher Scientific (Scoresby, Australia) and medical grade pressure sensitive adhesive (PSA) tape ARcare 90445 was acquired from Adhesive Research (Glen Rock, PA, USA). The acrylic sheets were purchased from Plastics Center (Cheltenham, Australia). APTES and glutaraldehyde were prepared to 2% solution in ethanol and 2.5% solution in milli-Q water respectively. Both were filtered using a 0.22 µm membrane to remove large debris.

### 2.1. Sensor Fabrication

**Figure 1:**
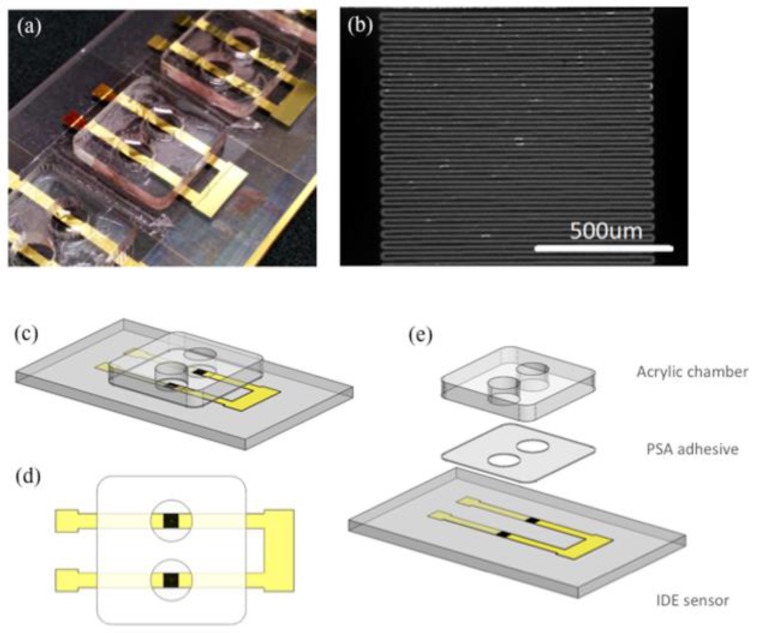
(**a**) An array of interdigitated electrode sensors on a microscope slide with acrylic detection chambers adhered; (**b**) A magnified image of the sensing region (scale bar 500 µm); (**c**) 3-D Illustration of a pair of interdigitated electrode (IDE) sensors; (**d**) Top view of the IDE sensor pair; (**e**) An exploded view of the IDE sensor pair showing different layers in correct order. From bottom to top: Glass slide (1 mm), laser ablated Cr/Au sensors (105 nm), patterned pressure sensitive adhesive tape (80 µm), acrylic well (1.5 mm).

Microscope glass slides were coated with 5 nm chromium (Cr) and 100 nm gold (Au) using electron beam evaporator (Intlvac Nanochrome II). The Cr/Au coated glass slides were then patterned using the laser ablation system (SUSS SLP300 with solid-state laser technology at 355 nm wavelength) to produce the microelectrode array. Figure 1a shows an array of sensors fabricated on the same microscope glass slide. Figure 1b illustrates the IDE sensing area with 49 electrodes. The width and length of the electrodes are 20 µm and 1 mm respectively. The gap between adjacent electrodes is 10 µm. Prior to sensor functionalization, the Cr/Au coated glass slides were treated with oxygen plasma before a layer of 10 nm thick SiO_2_ was selectively evaporated on the sensor surface.

### 2.2. Sensor Functionalization, Sample Preparation and Measurement Workflow

**Figure 2:**
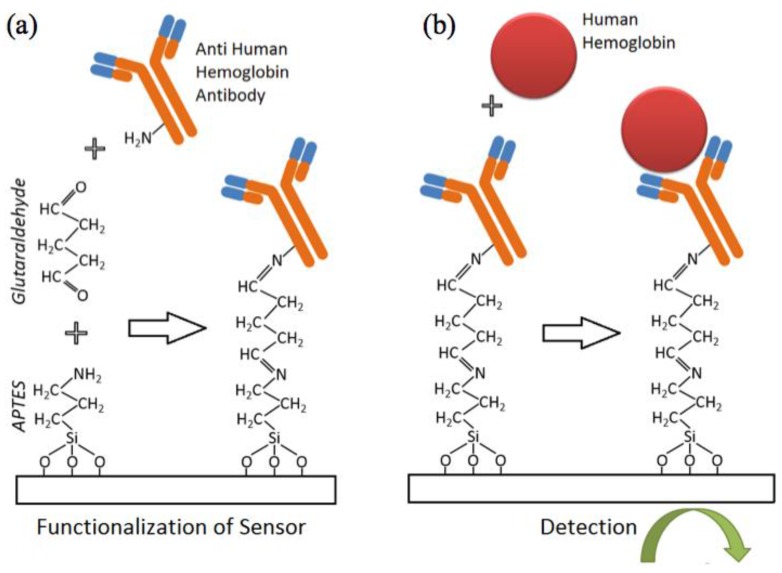
Functionalization protocol for the detection of hemoglobin; (**a**) Sensors are functionalized with APTES and glutaraldehyde prior to attachment of antibody. (**b**) Detection of target peformed electrically following solid-state Ab-Ag binding.

The fabricated IDE sensors were then prepared for functionalization and subsequent hemoglobin detection (Figure 2). Firstly, the sensors are cleaned thoroughly with acetone, iso-propanol and water (5 min each in ultra-sonicator bath). They were then dried under nitrogen stream followed by low power oxygen plasma treatment to activate the sensor surface with hydroxyl (–OH) groups. In detail, the glass slide with IDE sensors was immersed in 2% APTES in 95% ethanol for 1 h to allow for the aqueous silanization of the oxide surface to occur. This aqueous silanization process has been extensively described elsewhere [36] and has been implemented for various sensing applications [37,38]. The slide was then washed thoroughly 3 times in ethanol (5 min each) before it was incubated in 2.5% glutaraldehyde in milli-Q water for 2 h. This created aldehyde groups (–COH) on the sensor surface. The chip was then washed with milli-Q water before being dried in a fume hood. To isolate sensing regions from each other, acrylic wells with patterned PSA tape were quickly laminated on the slide surface. Figure 1c–e illustrates this process. 

Antibodies to human hemoglobin were immobilized on the sensor surface by spotting 20 µL of antibody solution (whole antiserum diluted 1:100 in 1× PBS) to each acrylic well. Primary amines groups (–NH_2_) (either on lysine residues or the N-terminus of each polypeptide chain of the antibody) facilitated the reaction with the exposed aldehyde groups available on the sensor surface. The antibody receptor was covalently immobilized on the sensor surface. Next, the glass slide was incubated in a humid chamber at room temperature for 30 min before being placed in a 4 °C refrigerator overnight. The incubated sensors were then gently washed with PBS and immersed in a blocking solution for half an hour (1% ethanolamine and 1% goat serum in 1× PBS). This step helped improve the specificity of the assay since ethanolamine blocks the unreacted aldehyde group and goat serum reduces non-specific antibody-antigen binding. After a gentle wash with 1× PBS, the sensor array was ready for testing.

Fresh human stool was collected and stored at 4 °C prior to usage, and then diluted in 1× PBS to a stock concentration of 10 mg/mL. Stock hemoglobin solution at 4 mg/mL was prepared by diluting human hemoglobin protein (in powder form) in 1× PBS. The stock hemoglobin solution was then diluted with stock fecal sample solution into testing concentrations of 0.01 mg, 4 mg, and 40 mg of hemoglobin per gram of fecal sample (in 1× PBS). Twenty microliters of the hemoglobin-spiked fecal samples were spotted on the sensors and incubated for an hour at room temperature in a wet chamber. 1× PBS was added as the negative control. After incubation, the sensor array was washed three times with PBS. Electrical measurements were then performed with the sensors immersed in PBS. 

### 2.3. Electrical Measurement Setup and Circuit Modelling

**Figure 3:**
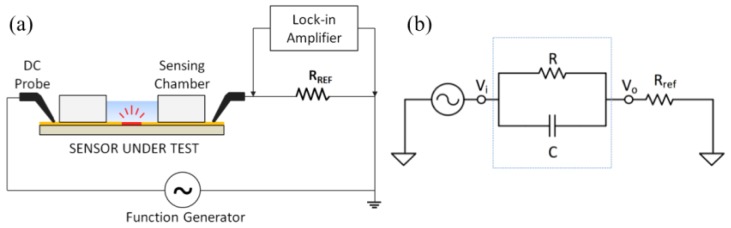
(**a**) Electrical measurement setup showing the sensor under test connected in series with a reference resistor (*R_ref_*). (**b**) Equivalent circuit of the experimental setup.

The IDE sensor was connected in series with a reference resistor *R_ref_* = 1 kΩ. The circuit (Figure 3) was excited by a sinusoid signal (peak-to-peak amplitude of *Vpp* = 100 mV) at a distinct set of frequencies (100 Hz, 1 kHz and 10 kHz) using a function generator. A lock-in amplifier setup utilizing the SR830 lock-in amplifier (Stanford Research System) was employed to measure the voltage on the reference resistor (*V*_0_) before and after the incubation of human hemoglobin protein. The changes in the amplitude and the phase of *V*_0_ versus hemoglobin concentrations at different frequencies were recorded and analyzed.

The IDE sensor was modeled as a resistor in parallel with a capacitor. These two components are connected in series with a reference resistor. The measured voltage $V_{0}$ is related to the signal generator output as

(1) $$V_{0}/V_{i}=R_{ref}/\left(Z_{s}+R_{ref}\right)$$

Here, *V_i_* in the input voltage, *R_ref_* is the resistance of the reference resistor, *Z_s_* is the impedance of the sensor, *R* is the resistance of the sensor. Moreover, the impedance properties are described in

(2) $$Z_{s}=R/\left(1+j\omega RC\right)$$

where *C* is the capacitance of the sensor and *ω* is the frequency in rad/s. By solving Equations (1) and (2), *R* and *C* can be calculated.

### 2.4. Statistical Analysis

Kruskal-Wallis analysis was performed for each applied frequency to determine the frequency most optimal for distinction of hemoglobin concentration. Prism for Mac was used for all statistical analyses. *p* < 0.05 was considered statistically significant. 

## 3. Results and Discussion

In this study, IDE sensors coated with a thin layer of SiO_2_ functionalized with antibodies to human hemoglobin were used to detect the hemoglobin in human fecal samples at very low concentration. The SiO_2_ coating helped to enhance the sensor sensitivity by reducing the internal double layer capacitance at the PBS/electrode interface as well as the polarization of the electrodes [37]. The output voltages measured at the reference resistor at 100 Hz, 1 kHz and 10 kHz frequencies (both amplitude and phase) were recorded. Baseline measurements were made just before the sample was placed on the functionalized sensors immersed in 20 µL of PBS. After 1-hour incubation of the sample on the sensor, the sensors were gently washed with PBS (3 × 5 min each) and measurements were performed again with the sensors immersed in 20 µL of PBS. The change in the output voltage (i.e., the difference in voltage across the reference resistor before and after the incubation of hemoglobin) was recorded using the lock-in amplifier. Detection of fecal hemoglobin was performed directly using the lock-in-amplifier setup, as well as indirectly by extracting the resistance and capacitance data.

**Figure 4:**
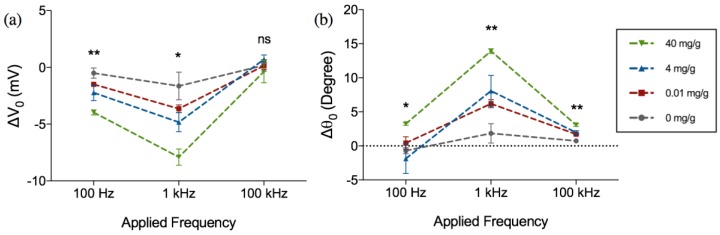
Frequency dependent change in (**a**) amplitude (Δ*V*_0_) and (**b**) phase (Δ*θ*_0_) of the output voltage after 1 h incubation in hemoglobin-spiked fecal samples of different concentrations. Figure represents mean with standard error of measurement, * indicates *p* < 0.05 and ** indicates *p* < 0.01, ns = not significant (Kruskal-Wallis ANOVA), *n* = 3 per concentration.

The amplitude and phase of this change (Δ*V*_0_ and Δ*θ*_0_), are shown in Figure 4 for different frequencies (100 Hz, 1 kHz and 10 kHz) as a function of varying hemoglobin concentrations (0.01, 4 and 40 mg·Hb/g·feces). As can be seen from the Figure 4a,b, the Δ*V*_0_ exhibits an increase in magnitude and an increase in Δ*θ*_0_ at the frequencies under consideration (negative Δ*V*_0_ and positive Δ*θ*_0_). It can also be observed that Δ*V*_0_ and Δ*θ*_0_ are not only most distinct at 1 kHz (*p* = 0.0205 for amplitude, *p* = 0.0014 for phase) and 100 Hz (*p* = 0.0028 for magnitude, *p* = 0.0328 for phase), but also exhibit the largest magnitude of change compared to the two other frequencies.

**Figure 5:**
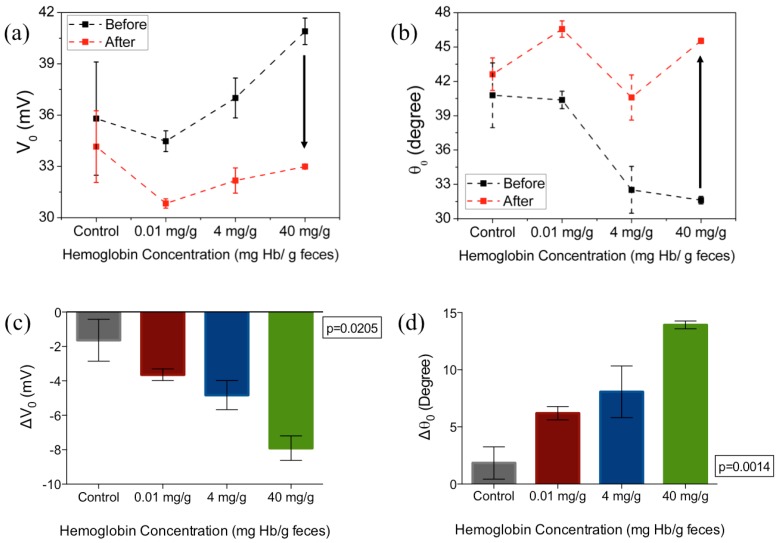
The (**a**) amplitude and (**b**) phase of the output voltage measured before and after 1 h incubation in hemoglobin-spiked fecal samples of different sample concentrations; (**c**,**d**) show the changes in amplitude and phase from baseline, respectively. Figures represent mean with standard error of the measurement. The excitation frequency is 1 kHz, *n*= 3 per concentration tested.

Figure 5a,b shows the amplitude (*V*_0_) and the phase (*θ*_0_) of the output voltage at the optimal frequency of 1 kHz. The arrows in Figure 5a,b indicates the direction of changes from before incubation of samples to after incubation and wash. The change in *V*_0_ and *θ*_0_ for varying hemoglobin concentration are plotted in Figure 5c, d respectively, at the optimal frequency of 1 kHz. At this set frequency, Δ*V*_0_ increased monotonically with the increase of hemoglobin concentration. At 0.01 mg·Hb/g, 4 mg/g and 40 mg/g, the average difference compared with control samples is 2 mV, 3.19 mV and 6.27 mV. The non-zero change in voltage in the control sample, when PBS was added to functionalized surface could be attributed to the effect of Helmholtz double layer at lower frequencies [38] while for samples with hemoglobin, the voltage changes are primarily due to the binding of the hemoglobin protein to receptor antibody tethered to the sensor surface. The increased change in voltage with hemoglobin concentrations indicates that the impedance of the biosensor increases with the higher concentrations of hemoglobin. 

**Figure 6:**
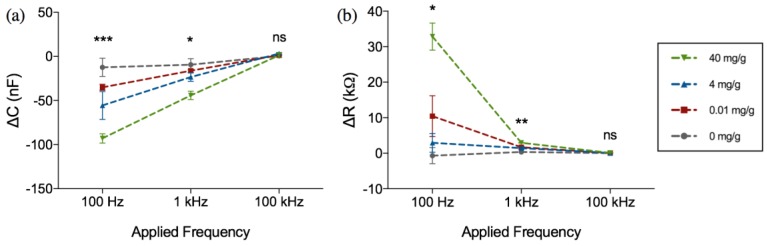
Frequency dependent change in (**a**) capacitance (Δ*C*) and (**b**) resistance (Δ*R*) after 1 h incubation in hemoglobin-spiked fecal samples of different concentrations (mg·Hb/g·feces). Figure represents mean with standard error of measurement, * indicates *p* < 0.05, ** indicates *p* < 0.01, *** indicates *p* < 0.001, ns = not significant (Kruskal-Wallis ANOVA), *n* = 3 per concentration.

The change in sensor impedance due to the binding of hemoglobin was further analyzed using the sensors’ equivalent resistance and capacitance calculated using Equations (1) and (2). Figure 6 shows the change in the resistance and capacitance at different frequencies and hemoglobin concentrations. As can be seen from Figure 6a,b, upon binding of hemoglobin to the surface immobilized receptor, the capacitance increases towards a negative value whilst the resistance increases towards a positive value. The change in resistance and capacitance was also highly dependent on the applied frequency.

**Figure 7:**
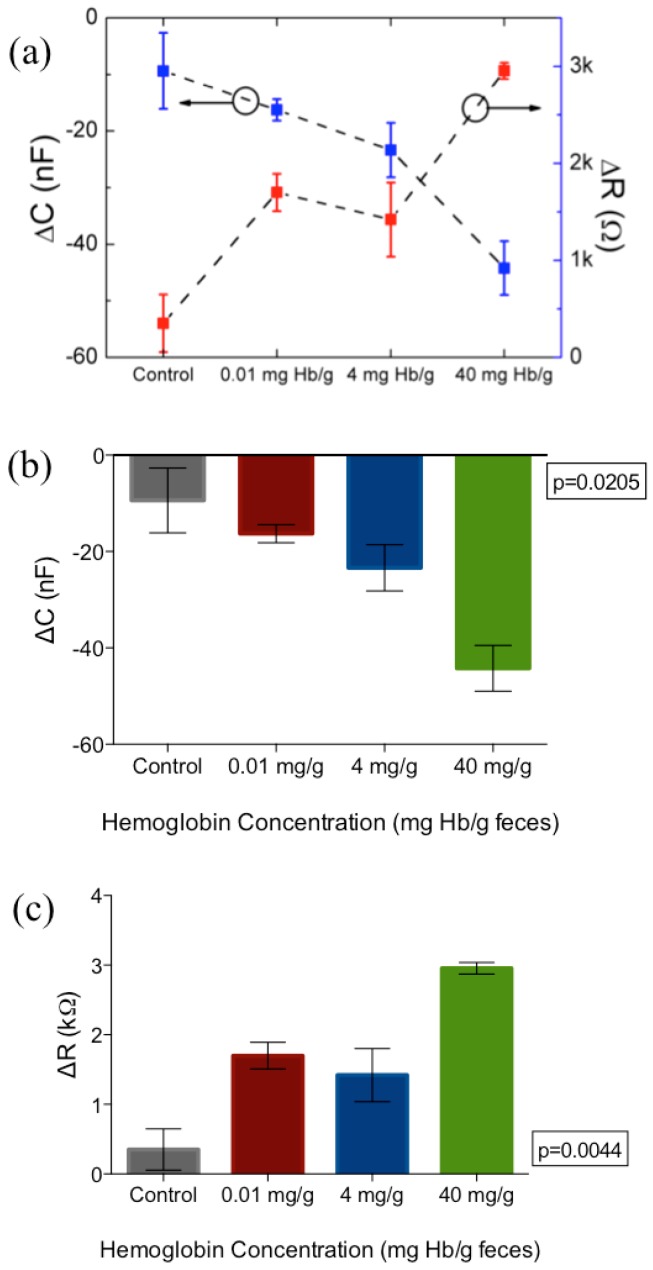
(**a**) Monotonic increase in resistance (red) and capacitance (blue) at 1 kHz with increasing concentration of fecal hemoglobin. (**b**,**c**) show changes from baseline for both capacitance and resistance respectively at 1 kHz applied frequency. Figure represents mean and standard error of measurement. N = 3 per concentration tested.

It was noted that the applied frequency affects both the magnitude of change as well as the distinction and consistency between samples. Although the magnitude of capacitance and resistance change is the largest at 100 Hz, it was observed that there was substantial overlap between the resistance values of 4 mg·Hb/g·Feces compared to negative control, as can be seen in Figure 6b. Because of the tendency of impedance drift to occur at lower frequencies such as 100 Hz and lower, more noise was observed at the 100 Hz frequency measurement. Therefore, 1 kHz was determined as the optimal frequency for parameters of capacitance and resistance. For more clarity, these changes in capacitance and resistance at 1 kHz are further extrapolated in Figure 7.

This result indicates a successful detection of feces with a LOD of 10 µg·Hb/g·feces. This study provides proof-of-concept data regarding the use of impedance biosensors as an alternative to current FIT tests. The results show that differentiation of various concentrations of hemoglobin in crude fecal samples can be performed in a rapid and label-free manner, using both direct lock-in amplifier parameters (amplitude and phase) as well as extracted impedance parameters (resistance and capacitance). By characterizing these parameters, detection can be optimized at a certain frequency, which in this study was found to be 1 kHz. Sample handling is crucial for the integration of the detection platform into a point-of-care device. This study shows that with minimal sample handling is required, with only PBS dilution involved to obtain characterization. Both minimal sample handling as well as label-free detection allows for reduced cost and time required for the detection platform.

## 4. Future Research

Future research is needed for the verification of our proof-of-principle findings in a larger panel of samples to determine the clinical sensitivity and specificity. Additional future directions of the work will include efforts to shorten the turnaround time and to integrate the platform into an automated system, which includes microfluidics and a built-in phase sensitive detection in the electronic reader that correlates reliably to the impedance changes occurring in the sensors. Because of the minimal sample processing required in this proof of concept stage, we envision that testing of the samples will only require a separate collection tube containing a set amount of buffer. Since the sensitivity and specificity of FIT for CRC diagnosis is dependent on its accurate measurement of hemoglobin in a given weight of feces, it will be important that fecal mass is consistent across tests. Therefore, it will be important to optimize different collection apparatus for consistent uptake of fecal mass.

## 5. Conclusions

The proposed sensors can detect the sample to a minimum concentration of 10 µg·Hb/g·feces, which is comparable to currently used bench-top quantitative FIT detection. Upon the introduction of hemoglobin protein spiked in human feces, the antibody receptors specifically bind to the proteins, causing a change in the device impedance within 1 h incubation. Differentiation between different hemoglobin concentrations in human fecal samples can be performed through several parameters either directly using the lock-in amplifier technique through amplitude and phase characterization, as well as indirectly by extracting equivalent values of capacitance and resistance. All parameters can be detected optimally at a single frequency of 1 kHz. The results presented in this paper provide proof-of-principle data that demonstrates the feasibility to quantitatively measure hemoglobin concentration in fecal sample using impedance spectroscopy technology without labelling, applicable for screening of colorectal cancer.

## Figures and Tables

**Table 1:** Comparison of Performance between Qualitative and Quantitative Fecal Immunochemical Test (FIT) platforms.

	Assay Platform	Physical Form	Clinical Cutoff	Quoted Lower Limit of Detection	Result Output
**Qualitative FIT**	Lateral-flow immune-chromatography	Cassette form	Varying depending on manufacture	ALL-DIAG-Hemotrust®: 6 µg·Hb/g·Feces [21]	Positive or Negative based on manufacturer cutoff
				Eiken OC Light®: 10 µg·Hb/g·Feces [22]	Subjective Interpretation
**Quantitative FIT**	Immuno-turbidimetric	Laboratory based, bulky machinery	Varying depending on end-user	HM-Jackarc®: 7 µg·Hb/g·Feces [23] NS-PLUS C15®: 4 µg·Hb/g·Feces [23]	Positive or Negative based on end user’s cutoff
				OC-SENSOR DIANA®: 10 µg·Hb/g·Feces [23] FOB Gold®: 2.55 µg·Hb/g·Feces [23]	Objective Interpretation
